# The Negative Relationship between Fouling Organisms and the Content of Eicosapentaenoic Acid and Docosahexaenoic Acid in Cultivated Pacific Oysters, *Crassostrea gigas*

**DOI:** 10.3390/md19070369

**Published:** 2021-06-25

**Authors:** Megumu Fujibayashi, Osamu Nishimura, Takashi Sakamaki

**Affiliations:** 1 Faculty of Engineering, Kyushu University, Fukuoka 819-0395, Japan; 2 Department of Civil and Environmental Engineering, Graduate School of Engineering, Tohoku University, Miyagi 980-8579, Japan; osamu.nishimura.d2@tohoku.ac.jp (O.N.); takashi.sakamaki.a5@tohoku.ac.jp (T.S.)

**Keywords:** dietary resource, *Mytilus galloprovincialis*, *Crassostrea gigas*, diatom, competition, biofouling, EPA, DHA

## Abstract

Bivalves serve as an important aquaculture product, as they are the source of essential fatty acids, such as eicosapentaenoic acid (EPA) and docosahexaenoic acid (DHA), in our diet. However, their cultivation in the wild can be affected by fouling organisms that, in turn, affect their EPA and DHA content. The effects of fouling organisms on the EPA and DHA contents of cultivated bivalves have not been well documented. We examined the effects of fouling organisms on the EPA and DHA contents and condition index of cultured oysters, *Crassostrea gigas*, in an aquaculture system. We sampled two-year-old oysters from five sites in Shizugawa Bay, Japan, in August 2014. Most of the fouling organisms were sponges, macroalgae, and *Mytilus galloprovincialis*. A significant negative relationship existed between the DHA content in *C. gigas* and the presence of sponges and macroalgae. A lower *C. gigas* EPA content corresponded to a higher *M. galloprovincialis* fouling mass and a lower *C. gigas* condition index. This can be explained by dietary competition between *C. gigas* and *M. galloprovincialis* for diatoms, which were the main producer of EPA in our study sites. Our findings indicate that fouling organisms likely reduce the EPA and DHA content in cultivated oysters. Therefore, our results suggest that the current efforts to remove fouling organisms from oyster clusters is an effective strategy to enhance the content of EPA and DHA in oysters.

## 1. Introduction

Bivalve aquaculture is common in coastal areas worldwide and is highly important for food production and ecosystem services [1]. In particular, oysters are a major bivalve aquaculture species; they comprised ~30% of global marine mollusc aquaculture in 2016 [2]. In general, shellfish aquaculture does not require artificial food supplements for the cultured organisms and is considered more environmentally friendly and sustainable than other feeding aquaculture species, such as finfish [3].

Bivalves are a rich source of highly unsaturated fatty acids, such as eicosapentaenoic acid (EPA) and docosahexaenoic acid (DHA) [4]. Consuming an adequate amount of these omega-3 fatty acids is important for human health because they have important roles in regulating biological functions [5,6]. These fatty acids are mainly synthesised by aquatic algae and are transferred to humans via the food chain [7,8]. However, due to the increasing demand for these essential fatty acids due to population growth, an estimation of the global supply of EPA and DHA for humans has indicated that adequate amounts of these fatty acids cannot be provided sustainably [9]. Therefore, the provision of EPA and DHA by aquaculture will become more important [4].

Biofouling is one of the most critical issues in suspended bivalve farming and substantially raises the costs of maintaining culture equipment and farming operations [10,11]. The biomass of fouling organisms devalues the products and increases the weight of culture equipment, creating difficulties in maintenance and farming operations [12]. Fouling organisms growing on bivalve shells are generally considered to negatively affect bivalve growth and survival [13,14] by weakening the water movement around the cultured bivalves and reducing the advective influx of food resources to them [12,15,16]. Moreover, numerical modelling studies on carrying capacity in bivalve aquaculture have demonstrated that food limitations owing to the overharvesting of the cultivated species can suppress growth of the cultivated species [17,18]. This implies that dietary competition with fouling suspension-feeding organisms may decrease the growth rate of these organisms. In fact, dietary competition has been highlighted as one of the main mechanisms of negative effects on the growth of cultivated species in laboratory experiments [19]. Since EPA and DHA are obtained from dietary sources, dietary competition may lead to a reduction of EPA and DHA in cultivated species. However, to our knowledge, no study has yet evaluated the effect of fouling organisms on the content of EPA and DHA in cultivated species.

*Crassostrea gigas* is one of the most economically important cultured oyster species in Northeast Asia [20]. Fouling marine organisms on oyster shells comprise various taxonomic groups, such as molluscs, bryozoans, barnacles, sponges, algae, ascidians, hydrozoans, and polychaetes [21,22,23]. Although the positive effects of fouling organisms on the growth of *C. gigas* have been reported in one case, fouling organisms can negatively affect the growth of *C. gigas* by food and space competition and require additional cost for maintenance to operators [23]. The objective of this study was to examine how fouling organisms affect the content of EPA and DHA in *C. gigas*. Furthermore, body condition, which is expressed as the relative weight of whole soft tissue to shell volume, is considered an important index of the product value of the cultivated oyster [24]. We collected oysters and fouling organisms from oyster farming sites in a temperate bay, located in Northeast Japan, and analysed the fatty acid composition and body condition of the *C. gigas* oysters.

## 2. Results

**Figure 1:**
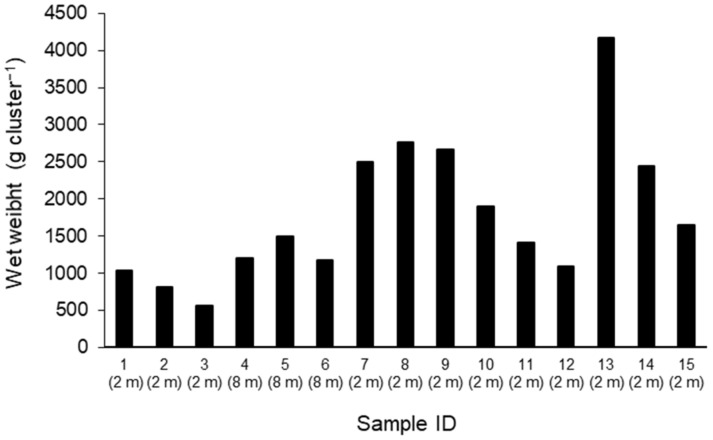
Total wet weight of fouling organisms of each sample. The values given in parentheses represent collecting water depth.

**Figure 2:**
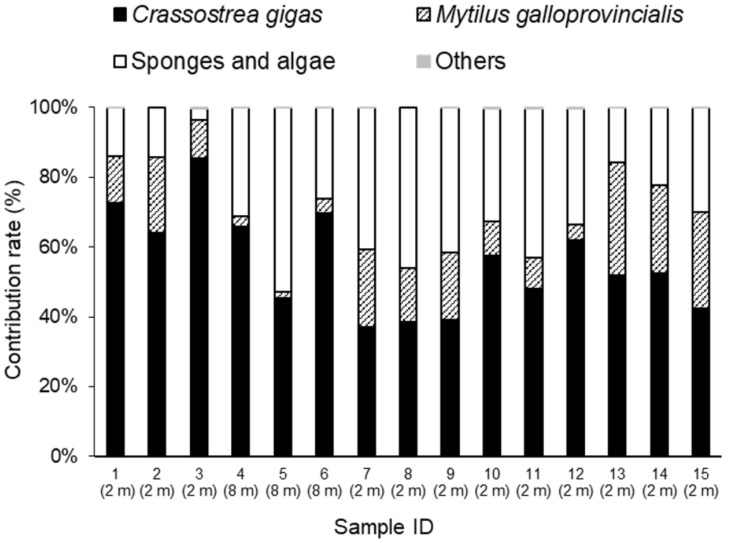
The contribution of each component within the sampled clusters from each sampling site. The “Other” component comprised mainly polychaetes, cirripedians, and decapod crustaceans. The mean values of *Crassostrea gigas*, *Mytilus galloprovincialis*, sponge and algae, and others are 55.5, 14.7, 29.7, and 0.1%, respectively. The values given in parentheses represent collecting water depth.

The wet weight of fouling organisms ranged from 567 to 4177 g cluster^−1^ (Figure 1), and the mean value was 1795 g cluster^−1^. The relative weight of fouling organisms by taxonomic group differed between the 15 sampled clusters (Figure 2). The major components were generally sponges and macroalgae, which contributed 3–53% of the total wet weight of the clusters, and the mean value was 29.7%. *M. galloprovincialis* was the second most dominant fouling organism, ranging from 2 to 32% of the total wet weight of the clusters, and the mean value was 14.7%. Other organisms, consisting of mainly polychaetes, cirripedians, and decapod crustaceans, made a minor contribution to the clusters in our study sites (below 0.5%).

**Figure 3:**
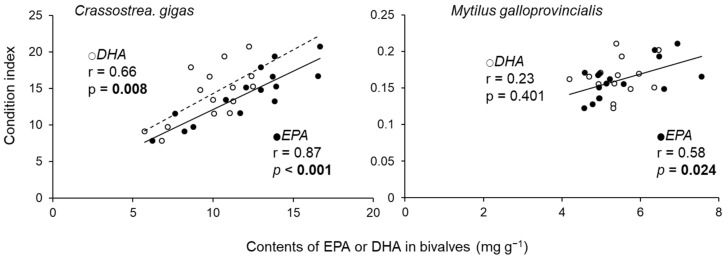
The relationship between the condition index and EPA or DHA content in *Crassostrea gigas* and *Mytilus galloprovincialis*. Each plot is the average of five individuals from each cluster.

**Figure 4:**
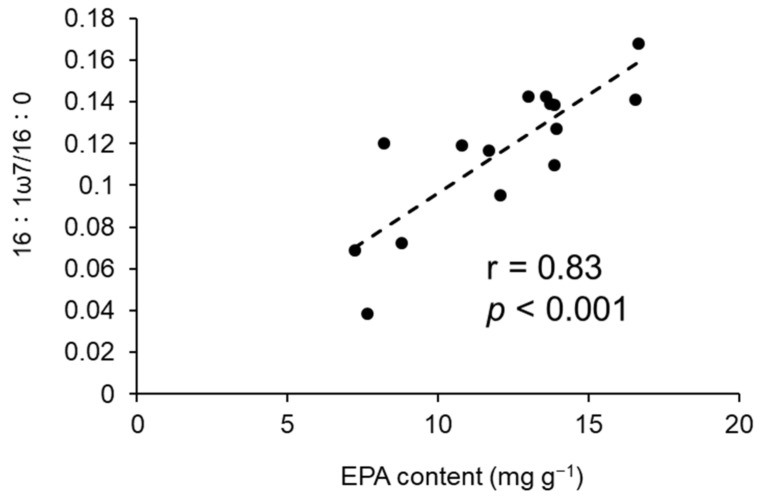
The correlation of the EPA content and the ratios of palmitoleic acid (16:1ω7) to palmitic acid (16:0) in *Crassostrea gigas*. Each plot is the average of five individuals from each cluster.

The condition index and content of EPA in *C. gigas* individuals were negatively correlated with the wet weight of *M. galloprovincialis* (Table 1). Negative correlations were also detected between the DHA content in *C. gigas* individuals and the wet weight of sponges and macroalgae (Table 1). For the relative weight of fouling organisms, *M. galloprovincialis* correlated negatively with EPA content in *C. gigas* individuals and CI (Table 1). Sponges and algae correlated negatively with the total wet weight, EPA with clusters, DHA with individuals, and DHA with clusters of *C. gigas*. The CI of *C. gigas* and *M. galloprovincialis* had a significant positive relationship with the EPA content (Figure 3). In addition, a significant positive relationship between CI and DHA was detected for *C. gigas* (Figure 3). The EPA content in *C. gigas* showed a significant positive relationship with the ratios of palmitoleic acid (16:1ω7) to palmitic acid (16:0) in *C. gigas* (Figure 4).

## 3. Discussion

The main fouling species of *C. gigas* aquaculture in the studied bay were sponges, algae, and *M. galloprovincialis* regardless of sampling site and depth, a finding that was partially inconsistent with some previous aquaculture studies conducted in Japan, which reported that mussels were dominant [25,26,27]. Mazouni et al. [28] reported that the predominant fouling organisms on *C. giga* clusters were ascidians. Royer et al. [29] reported that *C. gigas* clusters were mostly fouled by barnacles. Rodriguez et al. [22] found that ascidians, bryozoans, sponges, hydrozoans, and algae were the predominant colonisers on oyster farming beds, indicating that biofouling communities differ compositionally, even for the same host species [30]. This can be explained by the difference of environmental factors and climate among previous reports and the current study. Spatiotemporally variable factors, such as water temperature [22], the season [28], and larval supply from the benthic community [31], influence the settlement, abundance, and community structure in oyster farms.

While the negative effects of fouling organisms on oysters have been widely reported [23,32], some studies have indicated no significant effect of fouling organisms on cultivated oysters [29,33]. These inconsistent findings may indicate that the effects of fouling organisms on host oysters are species-specific and depend on focal factors (e.g., growth rate, survival rate, etc.) [30]. Similarly, in this study, the effects of sponges, macroalgae, and *M. galloprovincialis* on the fatty acid content and condition of *C. gigas* were different.

Sponges and macroalgal mixtures seem to have decreased the DHA content of *C. gigas* individuals. It is known that *C. gigas* have a poor ability to carry out biosynthesis of DHA [34,35]. Thus, the reduction of DHA in *C. gigas* implies that the dietary intake of DHA sources was reduced. DHA is abundant in some algal classes, such as dinoflagellates [36] and haptophyta [37]. For Shizugawa bay near farm A in July 2017, dinoflagellates were detected; however, haptophyta were not observed (Sakamaki, unpublished data, Table S1). Since sponges are suspension feeders [38], dietary competition for dinoflagellates between oysters and sponges is one of the possible mechanisms for the reduction of DHA in oysters. However, sponges generally ingest smaller particles, and their main dietary sources are known to be bacteria [39]. In addition, sponges can meet their dietary requirements through ingesting particles of <1 μm in diameter [40]. Therefore, competition is unlikely to explain the reduction of DHA in oysters, because most dinoflagellates are larger than 1 μm in diameter.

For macroalgae, allelopathy could be a possible mechanism for the reduction of DHA in oysters. Brown algae and *Ulva* spp., which were dominant macroalgae in our oyster aquaculture, have been demonstrated to significantly reduce the growth of dinoflagellates through allelopathy [41,42]. This hypothesis could explain the reduction of DHA in oysters if we assume that the allelopathy was more effective for dinoflagellates. In fact, species-specific allelopathy effects have been demonstrated, including *Ulva* and dinoflagellates [43]. However, further research is needed to clarify the mechanisms behind the DHA reduction in *C. gigas* when sponges and macroalgae act as fouling organisms.

A mixture of sponges and macroalgae also seem to have reduced the amount of EPA and DHA in *C. gigas* clusters. Since a negative relationship between the total weight of *C. gigas* in a cluster and the relative weight of the sponge and macroalgal mixture was detected, the observed reduction of EPA and DHA in the oyster clusters can be explained by the reduction of the total biomass of the oysters, as the relative weight of the sponges and macroalgae increased.

**Figure 5:**
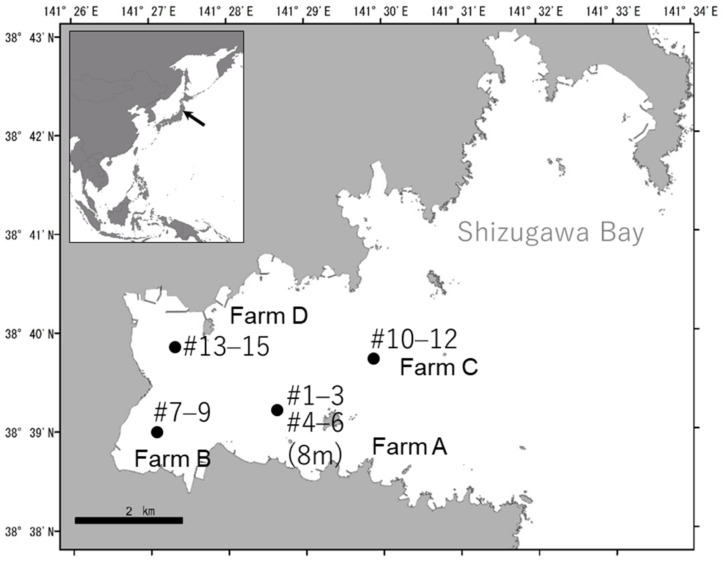
The location of sampling points in Shizugawa Bay, Japan.

*M. galloprovincialis* seem to have decreased the EPA content and CI of *C. gigas*. Although *C. gigas* can biosynthesise EPA from its precursors, its conversion efficiency is not enough to meet its requirement, and the EPA content in *C. gigas* mainly represents dietary EPA [35]. Thus, the amount of EPA in *C. gigas* seemed to depend mainly on their dietary intake, rather than on their own biosynthesis. Although EPA is abundant in diatoms, Cryptophyceae, and Rhodophyceae [36], the main EPA source for the assessed *C. gigas* was diatoms, because diatoms were dominant near our study sites (Sakamaki, unpublished data, Table S1). Furthermore, the observed significant positive relationship between EPA and 16:1n7/16:0 in oysters (Figure 5), which have been used as diatom markers, indicated that the main origin of EPA in the oysters were diatoms [44]. Pernet et al. [45] reported high bivalve growth rates during diatom bloom periods, and a feeding experiment by Piveteau et al. [46] also demonstrated an increase in the condition index of *C. gigas* when feeding on diatoms. In addition, a positive relationship was found between the EPA content and growth of a mussel species, *M. edulis* [47]. This was further demonstrated by the significant positive relationships between CI and EPA content in both bivalve species in our study. These findings support the idea that diatoms are a high-quality dietary source for both *C. gigas* and *M. galloprovincialis* and also indicate that there is probably a high competition potential for diatoms between *C. gigas* and *M. galloprovincialis*. Therefore, the condition index of *C. gigas* can be reduced as a result of competition with *M. galloprovincialis* for diatoms, with a consequent reduction of EPA acquisition.

Although these species have the potential to compete for diatoms, the CI of *M. galloprovincialis* was not negatively affected by the presence of *C. gigas*. This indicates that the dietary competition between *C. gigas* and *M. galloprovincialis* is not balanced. Although both *C. gigas* and *M. galloprovincialis* preferentially selected larger particles (>5 μm) in their diet, they did not necessarily need to compete, because *M. galloprovincialis* can also utilise smaller particles (<2 μm), which are not retained by *C. gigas* [48]. In fact, the invasion of *C. gigas* did not negatively affect local populations of the mussels *M. edulis* in Limfjord, Denmark, even though *C. gigas* were considered to have a competitive advantage owing to their higher filtration rate [49]. Contrary to this, our results clearly demonstrated that *M. galloprovincialis* has an advantage in dietary competition over *C. gigas*. There are two possible reasons for this. First, more than 97% of the EPA was distributed in particles of >2 μm near farm A in Shizugawa Bay [50], and dietary segregation by utilising small particles (<2 μm) by *M. galloprovincialis* was not valid in our study fields. The second reason was the vertical distribution of *C. gigas* and *M. galloprovincialis* in the cluster. *M. galloprovincialis* develops on the shells of *C. gigas* in Shizugawa Bay since oyster spats are artificially settled on the surface of scallop shells and grow before the scallop shells are put in the bay. As *M. galloprovincialis* settles on the surfaces of *C. gigas* shells, *M. galloprovincialis* has a spatial advantage in terms of feeding on diatoms before *C. gigas*. A portion of the diatoms ingested by the bivalves can survive [51], indicating that the faecal material of *M. galloprovincialis*, including diatoms, may supply the *C. gigas*, which are located inside the cluster. However, faecal material generally contains less EPA than suspended matter [50]. Therefore, *M. galloprovincialis* fouling on *C. gigas* could have substantial negative effects on the *C. gigas* in oyster aquaculture farms, in terms of EPA acquisition.

Although our data indicated that fouling organisms possibly reduce the EPA and DHA content of *C. gigas*, it should be noted that other environmental factors can also affect the EPA and DHA content of *C. gigas*. For instance, water temperature [52] and the reproductive cycle [53] are known to affect fatty acid profiles of oysters. In addition, the total amount and quality of supplied food sources, especially diatoms and dinoflagellate, may influence the EPA and DHA content in *C. gigas*. In this study, *C. gigas* were all collected with two-year-old oysters with similar sizes on the same day, which indicates that the effects of water temperature and the reproductive cycle can be assumed to not produce the difference of EPA and DHA content. Unfortunately, as we did not investigate the supplied food at each sampling point, further study is required to understand the effect of food availability. Furthermore, the fatty acid content of oysters changes spatially and seasonally [54,55], and this could be associated with composition and the amounts of fouling organisms. Therefore, long-term monitoring in different sites is an effective way for the comprehensive understanding of the effects of fouling organisms on the EPA and DHA content in cultivated species.

Although EPA and DHA are essential fatty acids for humans [5,6], the effect of fouling organisms on the EPA and DHA content in cultivated host species has not been comprehensively evaluated before. Our findings demonstrated a reduction of EPA and DHA in the cultivated oyster *C. gigas* likely due to fouling organisms. This can devalue the quality of the oysters as an aquaculture product. Removing fouling mussels is empirically known to reduce their negative impact on oyster growth [25,27]. Our results support the idea that the current efforts to remove fouling mussels from oyster clusters in the study region [56], which include hot water treatment and physical removal, are expected to enhance the content of EPA and DHA in the oysters.

## 4. Materials and Methods

### 4.1. Study Site

This study was conducted in the inner part of Shizugawa bay, located on the northeast side of Honshu Island, Japan (38.65° N, 141.50° E; Figure 5). The area of the bay is 46.8 km^2^, and the average and the maximum depth at the bay mouth is 30 and 54 m, respectively. In our observations in 2015, the annual range of seawater temperatures at a depth of 2 m was approximately 5–21 °C. The Pacific oyster *C. gigas* is one of the major aquaculture products in this bay, and longline oyster suspension facilities are distributed in the inner parts of the bay, in which the depth ranges from approximately 10–30 m. Oyster spats are artificially settled on scallop shells. Then, oyster clusters growing on scallop shells are tied at ~0.4 m intervals to ropes of approximately 8 to 10 m in length, and the ropes are vertically suspended from ~100 m longlines that are horizontally sustained by floating buoys. There are ~400 oyster farming longline facilities in the bay, which is based on the information provided by a local fishery cooperative.

### 4.2. Field Sampling

**Figure 6:**
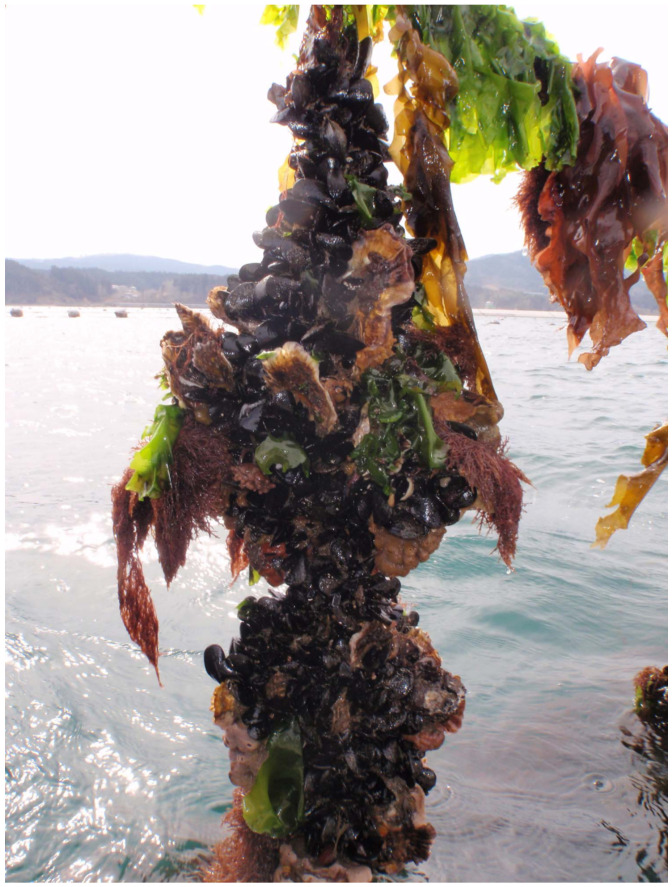
Two oyster clusters fouled by sponges, macroalgae, *M. galloprovincialis*, and other organisms. This photo was taken at farm B in February 2017.

To assess the composition of the fouling organism communities, oyster clusters growing on scallop shells were collected from four oyster farms in the inner part of the bay in August 2014 (Figure 5). The seawater temperature at a depth of 2 m in farm A was 20–21 °C. At all sampling farms, two-year-old oysters were cultured (the oysters were hatched in summer 2012). One oyster cluster (Figure 6) was collected from ~2 m depth from each of the three ropes that were randomly selected at each farm. Since vertical distribution of fouling organisms are expected to be different [23], three clusters were collected from ~8 m depth at farm A. Thus, 15 oyster clusters were sampled in total. Immediately after sample collection, the oyster clusters, including oysters, mussels, sponges and algae, and others, were disassembled by hand and sorted into four groups. Because sponges and macroalgae were attached together tightly and intricately, these two groups could not be separated. Thus, sponges and macroalgae were treated as one group. The macroalgae were mainly composed of *Ulva* and brown algae. Each group of sessile fouling organisms was measured for abundance and wet biomass. For the wet weight measurement, the samples were carefully dried with paper towels to minimise errors and then weighed using an electronic scale. For the sampled oysters, all individuals were measured for length, width, shell height, and whole-body wet weight. The soft tissues were then obtained by dissection and measured for wet weight. The shells were weighed after air drying. For each sampled cluster, to remove the effect of individual size on the fatty acid analysis, tissue samples were selected from five individuals with similar shell lengths (103.3 ± 12.3 mm, mean ± SD) and preserved in a freezer at −30 °C for later fatty acid analysis. Similarly, for the *M. galloprovincialis* mussels, which were predominant among the sessile fouling organisms, five individuals were randomly selected from each cluster sample with similar shell lengths (46.8 ± 6.8 mm, mean ± SD), and the shell size, wet weight of the soft tissue, and dry weight of the shell were measured. The soft tissue samples of the mussels were preserved in a freezer for later fatty acid analysis.

### 4.3. Fatty Acid Analysis

Since the whole body of the oyster is eaten by humans, we evaluated the fatty acid contents of oysters from the whole body. First, the soft tissue samples of the oysters and mussels were freeze-dried. Then, the whole body was powdered and homogenised in a blender. The ‘one-step method’ described by Abdulkadir and Tsuchiya [57] was applied for lipid extraction and derivatisation from the freeze-dried samples. Approximately 100 mg of freeze-dried sample was moved to a 50 mL glass tube. One millilitre of internal standard (1 mg tricosanoic acid per ml hexane), 4 mL hexane, and 2 mL 14% boron trifluoride methanol were added to the test tube, and nitrogen gas was added to fill the head space. The glass tubes were heated at 100 °C in a water bath for 2 h, then cooled to room temperature, and 1 mL hexane and 2 mL ultrapure water were added. The samples were shaken vigorously and centrifuged for 3 min at 2500 rpm (M-4000, KUBOTA Corp., Tokyo, Japan). The upper layer of hexane, which contained fatty acid methyl esters (FAME), was then placed in a 1.5 mL GC vial.

For quantification of the fatty acids, 1 µL FAME solution was injected into a gas chromatograph with an FID detector (GC-2014, Shimadzu, Kyoto, Japan) equipped with a capillary column (Select FAME, 100 m × 0.25 mm i.d., Agilent Technologies, Tokyo, Japan). The analytical conditions followed those outlined by Fujibayashi et al. [58]. The peak of each fatty acid was identified by comparison with the retention time of commercial standard mixtures (Supelco37, PUFA No.3, Bacterial FAME, Supelco^®^, Darmstadt, Germany). The amount of each fatty acid (milligram fatty acid per dry weight of animal) was calculated by following the method of Abdulkadir and Tsuchiya [57], with the internal standard (i.e., tricosanoic acid).

### 4.4. Data Analysis

We applied two condition indices (CI1 and CI2) in this study. CI1 and CI2 have been generally applied for oysters and other bivalve species, including mussels [24,59,60].

The oyster body condition index (CI1), which was developed by Lawrence and Scott [50], was calculated by

CI1 = (Dfw (g))/(Ww (g) − Shw (g)) × 100

where Dfw is the dry weight of soft tissue, which was measured after freeze drying, Ww is the total wet weight of the shell and soft tissue without any fouling organisms, and Shw is the dried shell weight. CI1 expresses the ratio of the dry weight of soft tissue to the internal shell volume, with the assumption that the density of soft tissue is almost the same as that of seawater. This assumption has been validated in oysters [24]. However, there has been no attempt to verify this for *M. galloprovincialis,* and Lucas and Beninger [59] pointed out that it is unlikely that the underlying assumptions are applicable to all bivalves. Therefore, we considered CI2 more appropriate for *M. galloprovincialis* in this study.

For mussels, the following condition index was applied [60]:

CI2 = (Dfw (g))/(Shw (g))

To examine the effects of fouling organisms on the CI and content of EPA and DHA in the cultivated oysters, correlation analysis was conducted by SPSS software (IBM, ver.20). All data were explored for normality using a Kolmogorov–Smirnov test and normality was not supported for the wet weight and relative weight of *M. galloprovincialis*. Then, Spearman rank correlation analysis was applied for *M. galloprovincialis*, and Pearson’s correlation analysis was applied for other fouling organisms. Fouling organisms were expressed as the total wet weight (g cluster^−^^1^). Furthermore, relative weight to oysters (g g^−1^) was also calculated since relative weight of fouling organisms can be expected to affect *C. gigas*. For the content of EPA and DHA, the concentration in each individual (mg g^−1^) and total amount in a cluster (g cluster^−1^) were evaluated and applied in the correlation analysis.

## Figures and Tables

**Table 1:** The r values of the correlation analysis between cultivated oysters and the main fouling organisms. Spearman rank correlation analysis was applied for *Mytilus galloprovincialis* and Pearson’s correlation analysis was applied for sponges and algae.

Fouling Organisms		*Crassostrea gigas*					
		Total Wet Weight	CI	EPA	DHA	EPA	DHA
	unit	g cluster^−1^	-	mg g^−1^		g cluster^−1^	
Wet weight							
*Mytilus galloprovincialis*	g cluster^−1^	0.204	−0.743 **	−0.689 **	−0.154	−0.250	−0.061
Sponges and algae	g cluster^−1^	−0.189	−0.409	−0.475	−0.642 **	−0.409	−0.357
Wet weight ratios to oyster							
*Mytilus galloprovincialis*	g g^−1^	−0.071	−0.661 **	−0.646 **	−0.310	−0.421	−0.236
Sponges and algae	g g^−1^	−0.649 **	−0.259	−0.312	−0.585 *	−0.697 **	−0.732 **

Bold represents a significant relationship, *: *p* < 0.05, **: *p* < 0.01.

## Data Availability

The data are included in the manuscript.

## Supplementary Materials

The following are available online at https://www.mdpi.com/article/10.3390/md19070369/s1, Table S1: Composition of phytoplankton taxa collected near farm A in July 2017 (cell L^−1^).
